# Propagation Analysis of an RFID System in the UHF Band in the Honeycomb Frame of a Beehive

**DOI:** 10.3390/s24113356

**Published:** 2024-05-23

**Authors:** José Lorenzo-López, Leandro Juan-Llácer

**Affiliations:** Department of Information and Communications Technology, Polytechnic University of Cartagena, 30202 Cartagena, Spain; jose.lorenzo@upct.es

**Keywords:** RFID, honeycomb, modelling, propagation

## Abstract

In recent years, communication systems, including RFID, have been used in intelligent beehives for beekeeping. RFID systems in the UHF frequency band offer reading distances of tens of centimetres, allowing the localisation and identification of the queen bee inside the hive. With this purpose, this work proposes an analysis of an environment of propagation that consists of a honeycomb frame, where the reader is placed within the frame, and the tag is placed in different positions over it. A honeycomb frame consists of a wooden box containing a honey wax panel, supported by metallic wires. The environment is modelled theoretically using its S-parameters and simulated in CST Studio. An analysis of these results and empirical measurements is performed. The results show that a periodicity in the received power of the tag is found with respect to the distance to the reader when the tag is located in a direction parallel to the wire, where local maximum and minimum values are found. Additionally, when the tag is placed over a wire of the frame, a higher received power is obtained compared to the case where the tag is placed between two wires. Furthermore, it has been observed that the reading range has increased with respect to free space, covering the full frame.

## 1. Introduction

Bees are known for their important role in plant pollination [1,2]. However, their population has been decreasing in recent times [3]. Thus, it is important for beekeepers to make sure that beehives are healthy and functioning correctly. One of the tasks necessary to accomplish this is to locate and identify the queen bee inside of the hive. Usually, this process involves visually inspecting all the honeycombs until the queen bee is located, and usually a drop of paint is placed on her thorax to facilitate the task [4]. The main disadvantage of this is that it can put the life of the queen at risk, as many honeycombs need to be manipulated.

Over the last few years, communication technologies have been introduced in beekeeping to facilitate the measurement and maintenance of beehives. Non-invasive systems have been developed in this context, such as that presented in [5], which uses sensors that measure parameters such as the humidity, the temperature, and the sound produced by the bees.

Radio frequency identification (RFID) systems have also been introduced to mark the bees. These systems consist of a reader and a tag. The reader sends a signal that activates the tag. Afterwards, the tags receive the signal and send a response from their internal memory [6] when they are inside the reading range [7]. Typical RFID systems used in intelligent beehives in the ultra-high-frequency (UHF) band provide measuring distances of up to tens of centimetres due to the size of the tag antenna. A range distance of up to one centimetre is enough for the system for counting bees developed in [8], where the bee is marked with a tag and reader antennas are located at the hive entrance. Reading ranges of tens of centimetres have been achieved, allowing the identification and location of the bees inside the beehive [9,10]. In this case, the reader is located externally, and the tag is placed on the bee, with dimensions on the order of millimetres.

A propagation analysis of the RFID system has been carried out, considering whether the system is in the far-field (FF) region [11] or the near-field (NF) region [12,13], according to the dimensions of the antennas used with respect to the wavelength. Additionally, an environment-independent model, valid for both the FF and NF, is proposed in [14]. When studying FF systems, the antenna gain concept is employed [11]. On the contrary, NF analysis requires a different approach, such as the model proposed in [12,13], which uses the concept of near-field magnetic flux. The model in [14] introduces the concept of transfer impedance, which characterises the effects of the environment as a function of the voltage and current in one of the antennas, without assuming FF or NF regions.

In this work, a propagation analysis of an RFID system in the UHF band in the honeycomb frame of a beehive, which consists of a wooden box containing a honey wax panel, supported by metallic wires, has been performed. The system (reader, frame, and tag) has been modelled using its S-parameters, without assuming FF or NF regions, as in [14]. The received power in the tag is obtained directly from the power transmitted in the reader and the S-parameters. Simulation software, such as CST Studio Suite 2023 (Johnston, RI, USA), provides S-parameter calculations from a 3D model of the environment. The analysis includes comparisons between the theoretical model and empirical measurements.

The paper is structured as follows. In Section 2, the propagation environment is detailed, as well as the RFID system employed and the methodology. Section 3 shows the obtained results, and the main conclusions are described in Section 4.

## 2. Propagation Environment and Measuring System

In this section, the environment considered for the propagation analysis, as well as the methodology and system employed, is detailed. For each element described, its equivalent, modelled in CST Studio, is shown.

### 2.1. Propagation Environment

**Figure 1:**
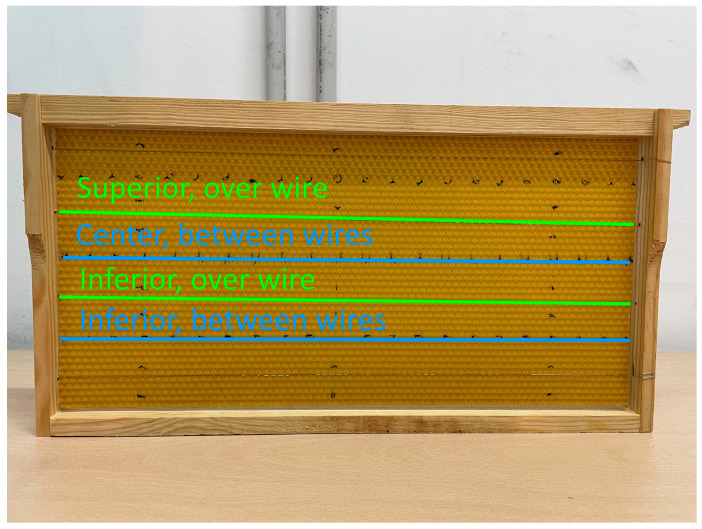
Considered honeycomb frame, with zones of interest.

The most common beehives employed in beekeeping are the Dadant, or industrial beehives. These hives consist of a series of honeycombs. A Dadant honeycomb, or frame, is composed of a wooden box that holds the entire structure and a set of parallel wires that are arranged horizontally and attached to the wood. Then, a thin layer of beeswax is placed inside the frame, and it is held by the wires. This layer of beeswax helps the bees to build their hexagonal honeycombs. In this work, each of the frames has external dimensions of 44.5 cm × 23.5 cm, and a thickness of 2 cm. The layer of honey wax is 42.5 cm × 20.5 cm, with a thickness on the order of 1 mm. Finally, it has four wires separated by 5.66 cm and joined at their ends. A front view can be seen in Figure 1.

This environment has been modelled theoretically using CST Studio. This program allows the simulation of electromagnetic environments from structure design and the analysis of relevant parameters, such as S-parameters, electric and magnetic fields, etc. The frame in Figure 1 was modelled in CST Studio, considering the dimensions already mentioned, employing materials that are available by default in the program for the wood and steel wires.

To model the honey wax, a new material was defined, with a relative permittivity of 3, which was selected arbitrarily.

**Figure 2:**
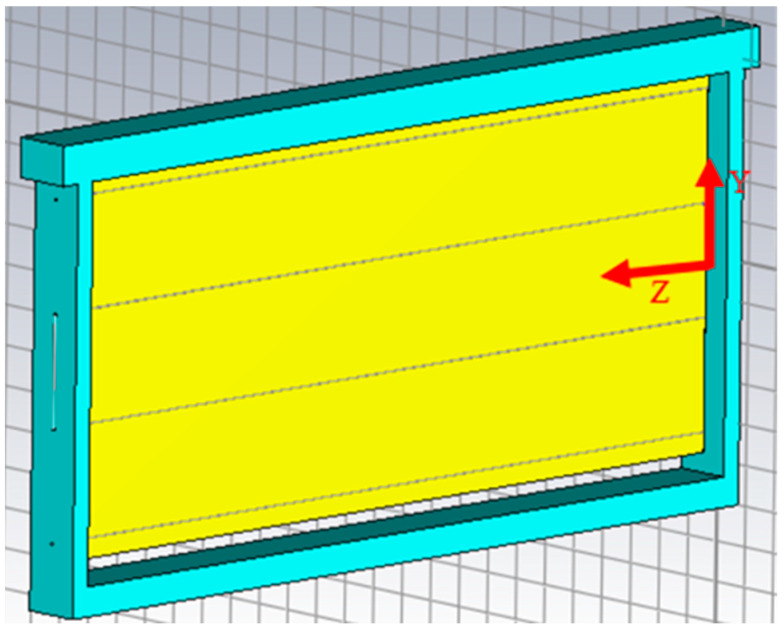
Modelling of a honeycomb frame in CST Studio.

All materials will be considered lossless to improve simulation time. The resulting model is shown in Figure 2.

### 2.2. Measuring System

**Figure 3:**
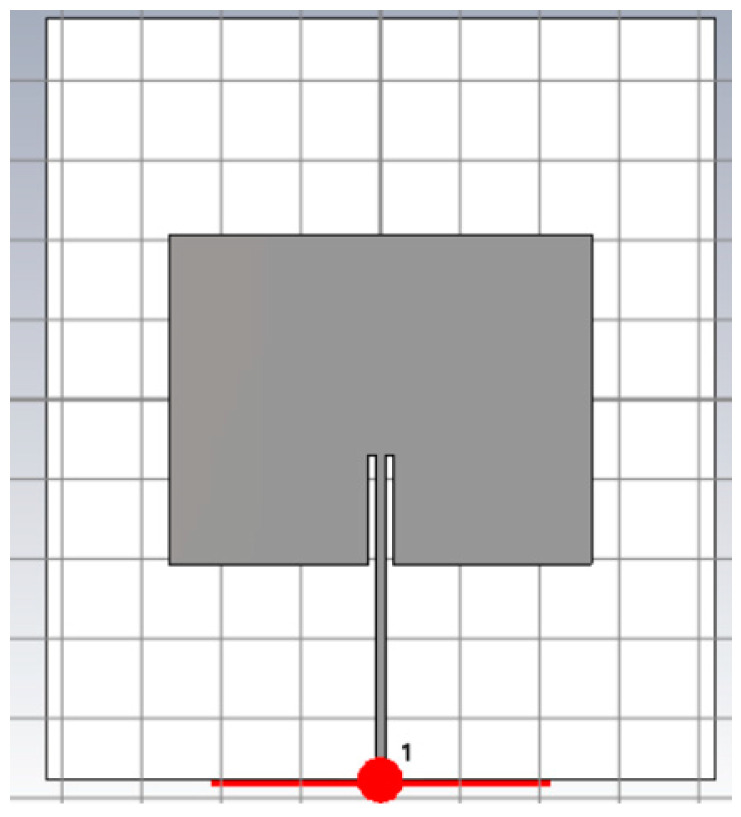
Reader antenna modelled in CST Studio.

The measuring system consists of an RFID reader and a tag, matched to the working frequency of 868 MHz. The reader is composed of a series of modules governed by a microcontroller, connected to an antenna. The reader antenna is a Keonn SP-11 (Keonn Technologies, Barcelona, Spain) [15], the reader module is a CAEN Hadron (CAEN, Viareggio, Italy) [16], and the microcontroller is an ESP32 (Espressif Systems, Shanghai, China) [17]. To model the reading antenna, a patch antenna was designed, with dimensions of 10.68 × 8.28 cm, on an FR4 substrate. This configuration can be seen in Figure 3.

**Figure 4:**
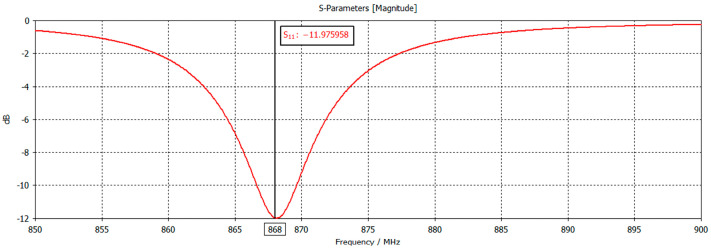
Modelled reader antenna s_11_ parameter with respect to frequency, measured in CST Studio.

The reader antenna was designed to be matched at 868 MHz, as observed in its s_11_ parameter, shown in Figure 4.

The tag consists of a chip and a micro-antenna. This micro-antenna is matched to the load impedance, in this case an Impinj Monza R6 chip (Impinj, Seattle, WA, USA) [18], along with the LXMS21ACMF-218 (Mouser Electronics, Inc., Munich, Germany) data transponder [19]. The tag antenna has a circular polarization, consisting of a two-layer loop design [9]. The queen bee moves over the beeswax with the tag placed over its thorax, so we assume that the two-layer loop is in the YZ plane, parallel to the frame. Similarly to the reader antenna, the tag micro-antenna was also modelled in CST Studio [9].

**Figure 5:**
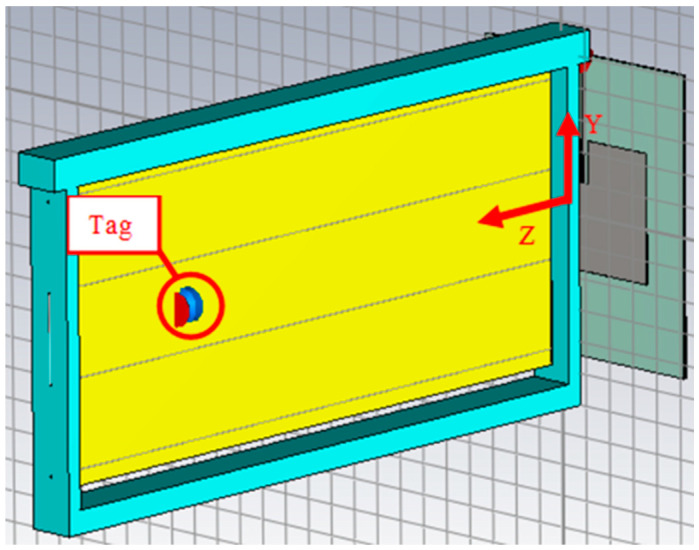
The total modelled system in CST Studio.

The total system, modelled in CST Studio to perform the simulations, is shown in Figure 5.

### 2.3. Methodology Employed

**Figure 6:**
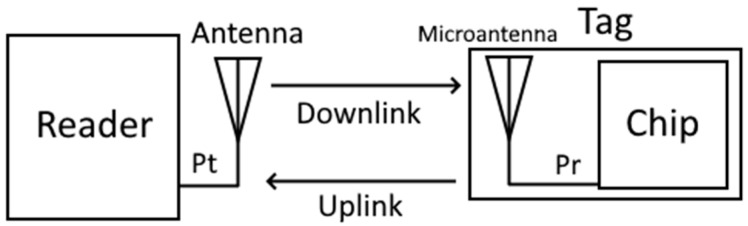
General diagram of the system.

In order to make tag-to-reader communication possible in an RFID system, two conditions must be met. On the one hand, a certain power from the reader must be received at the tag (downlink), and this must be higher than or equal to the minimum activation power of the tag’s chip. On the other hand, the signal that reaches the tag must reradiate back a certain power that, when it reaches the reader (uplink), must be higher than or equal to the reader’s sensitivity. A diagram of the system can be seen in Figure 6.

**Figure 7:**
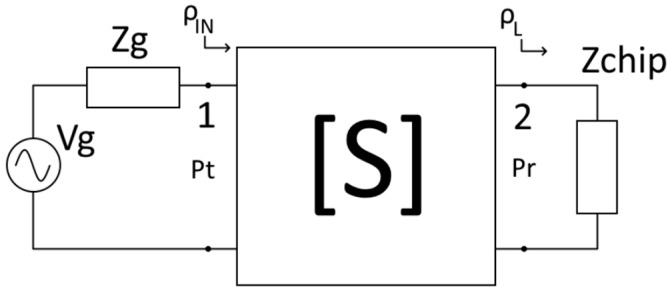
Circuit model of the system.

The environment can be modelled by its S-parameters, as shown in Figure 7. Port 1 is located in the reader antenna input and Port 2 is located in the tag’s micro-antenna output, where the chip with an impedance Z_chip_ is placed.

We can relate the transmitted power P_t_ and the received power P_r_ as follows [20]:

(1) $$\frac{P_{r}}{P_{t}}=\frac{{\left|s_{21}\right|}^{2}\left(1-{\left|\rho_{L}\right|}^{2}\right)}{\left(1-{\left|\rho_{\mathrm{IN}}\right|}^{2}\right){\left|1-s_{22}\rho_{L}\right|}^{2}}$$

Furthermore, if we observe the s_11_ parameter of the reader antenna in CST Studio (Figure 4), we can see that we have a good match at 868 MHz. Then, we can consider

(2) $$\rho_{\mathrm{IN}}\approx 0$$

Afterwards, we substitute (2) into (1) to obtain

(3) $$\frac{P_{r}}{P_{t}}=\frac{{\left|s_{21}\right|}^{2}\left(1-{\left|\rho_{L}\right|}^{2}\right)}{{\left|1-\rho_{L}\right|}^{2}}$$

Finally, we get

(4) $$P_{r}=\frac{P_{t}{\left|s_{21}\right|}^{2}\left(1-{\left|\rho_{L}\right|}^{2}\right)}{{\left|1-s_{22}\rho_{L}\right|}^{2}}$$

where the reflection coefficient of the load, $\rho_{L}$, is defined as

(5) $$\rho_{L}=\frac{Z_{\mathrm{chip}}-Z_{0}}{Z_{\mathrm{chip}}+Z_{0}}$$

where Z_chip_ is the chip impedance and Z_0_ is the reference impedance of the ports, which in this case is 50 Ohm. Then, we can compute the received power from the s_21_ and s_22_ parameters, which are obtainable through CST Studio. The procedure consists of executing the simulation and measuring the complex values of both parameters at 868 MHz for each distance on the *z*-axis (from reader to tag) and *y*-axis (based on whether the tag is over a wire or between them, as shown in Figure 1).

Fort the real measurements, a script was employed. This script returns the values of the relative signal strength indicator (RSSI) of a set of points, which are the same points considered for the CST simulation. The RSSI value indicates the quality of the reader-tag communication. This parameter, with values ranging from −40 to −80 dBm, fluctuates according to the channel conditions. When this parameter is detected, the power received at the tag is higher than or equal to the activation sensitivity of the chip. Specifically, the employed chip has a sensitivity of −22 dBm [18], and the reader applies a transmission power of 31.5 dBm [16].

The environment analysis is carried out by comparing the received power calculated with (4) from the S-parameters obtained in the CST Studio simulation with the RSSI measurements in the reader, placing the tag in the same points for both situations. These points have 2 cm between them on the *z*-axis. Four cases have been considered, and they are shown in Figure 1: placing the tag between two wires at the centre of the structure (centre, between wires); placing the tag between two wires in the lower position of the former case (inferior, between wires); placing the tag over the superior wire, second from the top to the bottom (superior, over wire); placing the tag over the inferior wire, third from the top to the bottom (inferior, over wire).

**Figure 8:**
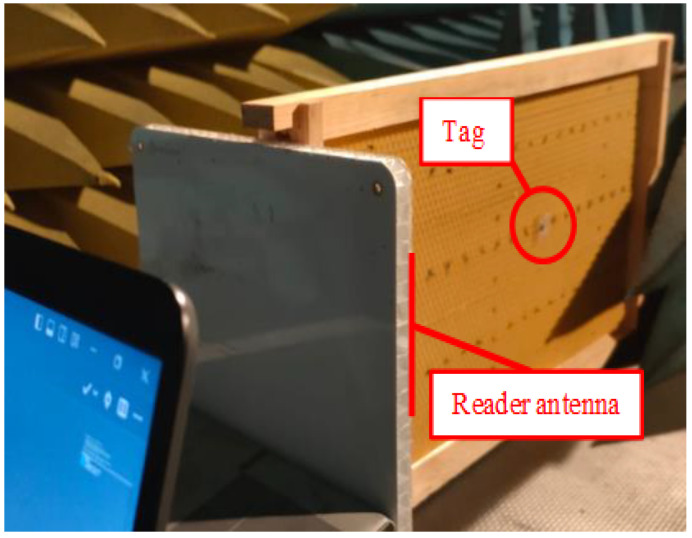
Measurement procedure.

RSSI measurements were carried out in an anechoic chamber, with the tag placed at the mentioned points. In Figure 8, an example of the procedure can be observed.

## 3. Results

The RSSI measurements results, compared to the power received obtained from (4), are shown below. The reference impedance in the ports, Z_0_, is 50 Ohms, which is the same as that of the sources. The impedance of the chip Z_chip_ is 13.4 – j126 Ohm [18]. As stated in the previous sections, RSSI values have no direct dependency with the received power, but detecting a RSSI value implies that the power received at the chip was at least −22 dBm. This means that the downlink is the most restrictive link in RFID communication, as found in [21].

The following two subsections consist of placing the tag between two wires and placing the tag over a wire, as described in Figure 1.

### 3.1. Tag between Two Wires

**Figure 9:**
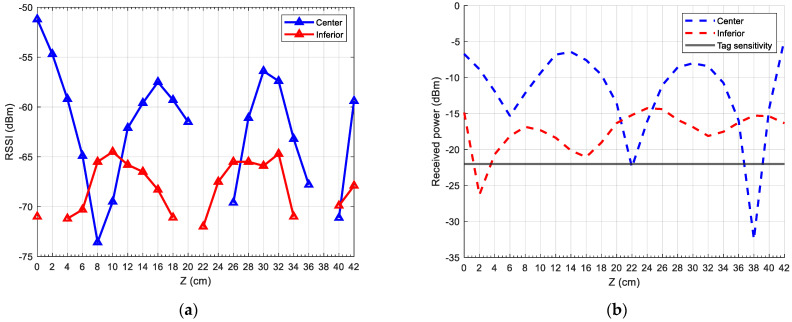
(**a**) Measured RSSI in the reader; (**b**) simulated received power in the tag between two wires.

In Figure 9a, we can see the RSSI measurements when the tag is placed between two wires. The RSSI values are expressed in dBm, and the reader-to-tag distances parallel to the wires are shown in centimetres. The blue points correspond to the superior position, and the red points to the inferior position, as shown in Figure 1. A series of zones where the RSSI value is null can be observed, specifically at 2, 20, 36, and 38 centimetres. As explained before, in these points, the tag received a power lower than its sensibility, so the tag was not detected.

The received power results simulated in CST, obtained with (4), are shown in Figure 9b. Three zones can be seen where the received power is at a relative minimum. These zones are located at approximately 2, 20, and 37 cm, which are close to the zones where the tag was not read. Additionally, the chip sensitivity of −22 dBm is shown in the figure.

For both figures, a certain periodicity can be observed in the behaviour of the RSSI and received power, as local minima are separated by a somewhat fixed distance, as well as the local maxima.

It is observed that, even though RSSI values do not have a direct relationship with power, the shapes of both figures are similar, but they seem slightly offset. Thus, the point where the tag is not detected is 20 cm according to the RSSI values, but we can see in the simulation that this point could be at 16 cm, where the received power is −21 dBm.

### 3.2. Tag over a Wire

**Figure 10:**
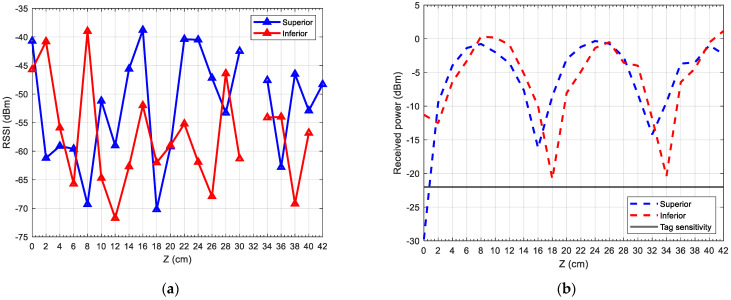
(**a**) Measured RSSI in the reader; (**b**) simulated received tag in the tag, over the wire.

In this case, Figure 10 is obtained; the RSSI values can be seen in Figure 10a, and the received simulated power from (4) is shown in Figure 10b.

In this case, the RSSI values have increased compared to placing the tag between two wires. We can also see that these values fluctuate much more than in the previous case. A possible explanation for this could be that the effect produced by the metallic wires is more unpredictable due to the reflections and interference that take place between them, as well as the RFID modulation effects not taken into account in the simulation. We observe that, according to the RSSI values, the tag is not detected only at 32 cm.

When it comes to the simulated received power, the minimum values are slightly offset with respect to the case where the tag was between two wires; they are approximately 0, 18, and 34 cm. The values of received power have also increased with respect to the case where the tag was between two wires in the simulation, but the periodicity observed previously is still present, as local minima and maxima seem to be separated by a fixed amount.

We can then conclude that placing the tag over a wire enhances the coverage of the system, even though the behaviour of the communication becomes more unpredictable.

While it is true that electromagnetic coupling is observed for both cases, when the tag is placed over the wires, this effect is significantly increased, as the tag antenna is nearer to the metallic structure formed by the wires.

On the other hand, in [9] it was observed that the RSSI values decreased with respect to the distance, until the tag was not detected, at 22 cm. However, in the frame, even though there are points where the tag is not detected, the reading range increases up to 42 cm.

## 4. Conclusions

First, a periodicity is observed in the behaviour of the received power with respect to the distance in a direction parallel to the wire. This produces a set of zones where the power received in the tag is higher than at other points closer to the reader. On the other hand, zones where the tag receives lower power are also produced, to the point where, in some cases, the tag cannot be detected.

In the second place, the coupling effect of the wires with the tag antenna is demonstrated. This effect is increased when the tag is placed over a wire, obtaining a higher average power compared to placing the tag between two wires, where the coupling effect is produced to a lesser extent. This means that placing the tag over the wires will provide a higher received power, although its RSSI behaviour will become more unpredictable. Furthermore, it has been observed that the reading range has increased with respect to free space, covering the full frame.

Future research is oriented towards analysing different parameters of the frame that were not taken into account in this paper, such as the electric permittivity of the beeswax, the thickness of the wires, and the number of wires in the frame. Moreover, a full hive could be analysed, extrapolating the results of a single frame to a 10-frame structure. On the other hand, theoretical studies can be performed to propose electromagnetic models that characterise the propagation of the system.

## Data Availability

The raw data supporting the conclusions of this article will be made available by the authors on request.
